# The role of phytochromes in regulating biosynthesis of sterol glycoalkaloid in eggplant leaves

**DOI:** 10.1371/journal.pone.0189481

**Published:** 2017-12-13

**Authors:** Cui-Cui Wang, Maria Sulli, Da-Qi Fu

**Affiliations:** 1 Fruit Biology Laboratory, College of Food Science and Nutritional Engineering, China Agricultural University, Beijing, China; 2 Italian National Agency for New Technologies, Energy and Sustainable Development, Roma, Italy; South China Agricultural University, CHINA

## Abstract

Glycoalkaloids are toxic compounds that are synthesized by many *Solanum* species. Glycoalkaloid biosynthesis is influenced by plant genetic and environmental conditions. Although many studies have shown that light is an important factor affecting glycoalkaloid biosynthesis, the specific mechanism is currently unknown. Chlorophyll and carotenoid biosynthesis depend on light signal transduction and share some intermediate metabolites with the glycoalkaloid biosynthetic pathway. Here, we used virus-induced gene silencing to silence genes encoding phytoene desaturase (PDS) and magnesium chelatase (CHLI and CHLH) to reduce chlorophyll and carotenoid levels in eggplant leaves. Quantification of carotenoid and chlorophyll levels is analyzed by LC/PDA/APCI/MS and semipolar metabolite profiling by LC/HESI/MS. Notably, the resulting lines showed decreases in glycoalkaloid production. We further found that the expression of some genes involved in the production of glycoalkaloids and other metabolites were suppressed in these silenced lines. Our results indicate that photosynthetic pigment accumulation affects steroidal glycoalkaloid biosynthesis in eggplant leaves. This finding lays the foundation for reducing the levels of endogenous antinutritional compounds in crops.

## Introduction

Steroidal glycoalkaloids (SGAs) are a class of nitrogenous secondary metabolites that are synthesized in many organs of Solanaceous plants including tomato (*Solanum lycopersicum*), potato (*Solanum tuberosum*) and eggplant (*Solanum melongena*) [1, 2]. SGAs inhibit the activity of acetylcholine enzymes and have a negative effect on the nervous and digestive systems of vertebrate animals [1]. Our demand for more and better food continues to increase. Improved nutritional qualities, as well as removal of antinutritional traits, are needed. Eggplant is an agronomically important vegetable, and is cultivated and consumed in many countries, which play an important role in the human diet. Therefore, the research on the biosynthesis of anti-nutrition glycoalkaloid is essential in eggplant. There have been many studies on the biosynthesis of glycoalkaloids in tomatoes and potatoes, due to their low contents and complicated metabolic pathways, few studies have focused on the regulation of SGA biosynthesis in eggplants.

Cholesterol is the proposed common precursor for the biosynthesis of both SGAs and steroidal saponins. Cholesterol is synthesized from desmosterol through the action of Sterol Side Chain Reductase 2 [3]. Glycoalkaloids have different structures in different Solanaceae plants (S1 Fig). A series of hydroxylation, oxidation and transamination reactions subsequently result in the biosynthesis of tomatidine in tomato and solanidine in potato [2], although the biosynthetic pathway of tomatidine, which differs from solanidine due to the presence of an F-ring, has not yet been resolved at the molecular level. The aglycones are glycosylated through the action of a series of glycosyltransferases [4] to form the final products dehydrotomatine and *α*-tomatine in tomato, *α*-solanine and *α*-chaconine in potato and *α*-solasonine and *α*-solamargine in eggplant [1, 2]. The eggplant compounds have the same F-ring as that of tomato, whereas their glycosyl moiety is more similar to that of potato glycoalkaloids. Glycoalkaloid biosynthesis in *Solanum* species is regulated by a group of clustered genes [5], and the biosynthesis of these compounds can be modulated by controlling the expression of these genes in the plant.

Glycoalkaloids usually accumulate to high levels in green plant tissues such as leaves, green fruits and green buds/sprouts [6]. Additionally, when potato tubers are exposed to sunlight and turn green, glycoalkaloid accumulation significantly increases compared to the control [7, 8].These findings suggest that glycoalkaloid biosynthesis might be associated with the presence of green pigments, such as chlorophyll and provide the theoretical basis for our experiments. Plant greening is a complex biological process, governed by environmental conditions and nuclear and plastid genes [9]. The chlorophyll, carotenoid and glycoalkaloid biosynthetic pathways share a common biosynthetic intermediate, isopentenyl pyrophosphate (IPP), indicating that the biosynthesis of each of these three compounds could influence that of the others (S2 Fig). However, the IPP required for SGA biosynthesis is synthesized in the cytoplasm via the mevalonate (MVA) pathway, while that required for the biosynthesis of carotenoids and of the phytol side chain of chlorophylls is synthesized in the chloroplast via the MEP pathway and the chlorophyll biosynthesis pathway, respectively.

In the current study, we explored how chlorophyll and carotenoid biosynthesis affect glycoalkaloid biosynthesis using eggplant. We blocked the synthesis of chlorophyll and carotenoids by silencing selected genes from their biosynthetic pathways via virus-induced gene silencing (VIGS) and investigated the resulting effects on SGA biosynthesis. Our results suggest that SGA biosynthesis depends on the accumulation of photosynthetic pigments, which opens an avenue for producing crops with reduced levels of endogenous antinutritional compounds.

## Material and methods

### Plant materials

Eggplant (*Solanum melongena* L. ‘Zhongnong changfeng’) was grown in the greenhouse under standard conditions (24–26°C under a 16 h light/8 h dark cycle). Eggplant seeds were germinated in plastic pots containing soil mixture. After one week, the seedlings were divided into two groups: one group was infiltrated with Agrobacterium strain GV3101 containing a silencing vector, while the other was treated with TRV (control).

### Silencing vector construction

The VIGS tool (http://solgenomics.net/tools/vigs) was used to design gene silencing regions to avoid off-target silencing, and the corresponding restriction enzyme site was added to the end of the primer: 500 bp *PDS*, 257 bp *ChlI* and 254 bp *ChlH* fragments were designed and amplified from tomato cDNA via PCR. The pTRV2-*SlPDS* pTRV2-*SlChlI* and pTRV2-*SlChlH* constructs were generated by inserting the PCR fragments into the pTRV2 vectors after both were digested with *Kpn*I and *Xho*I. The oligonucleotide primers used are listed in Supplementary S1 Table.

### *Agrobacterium tumefaciens* infiltration

**Fig 1:**
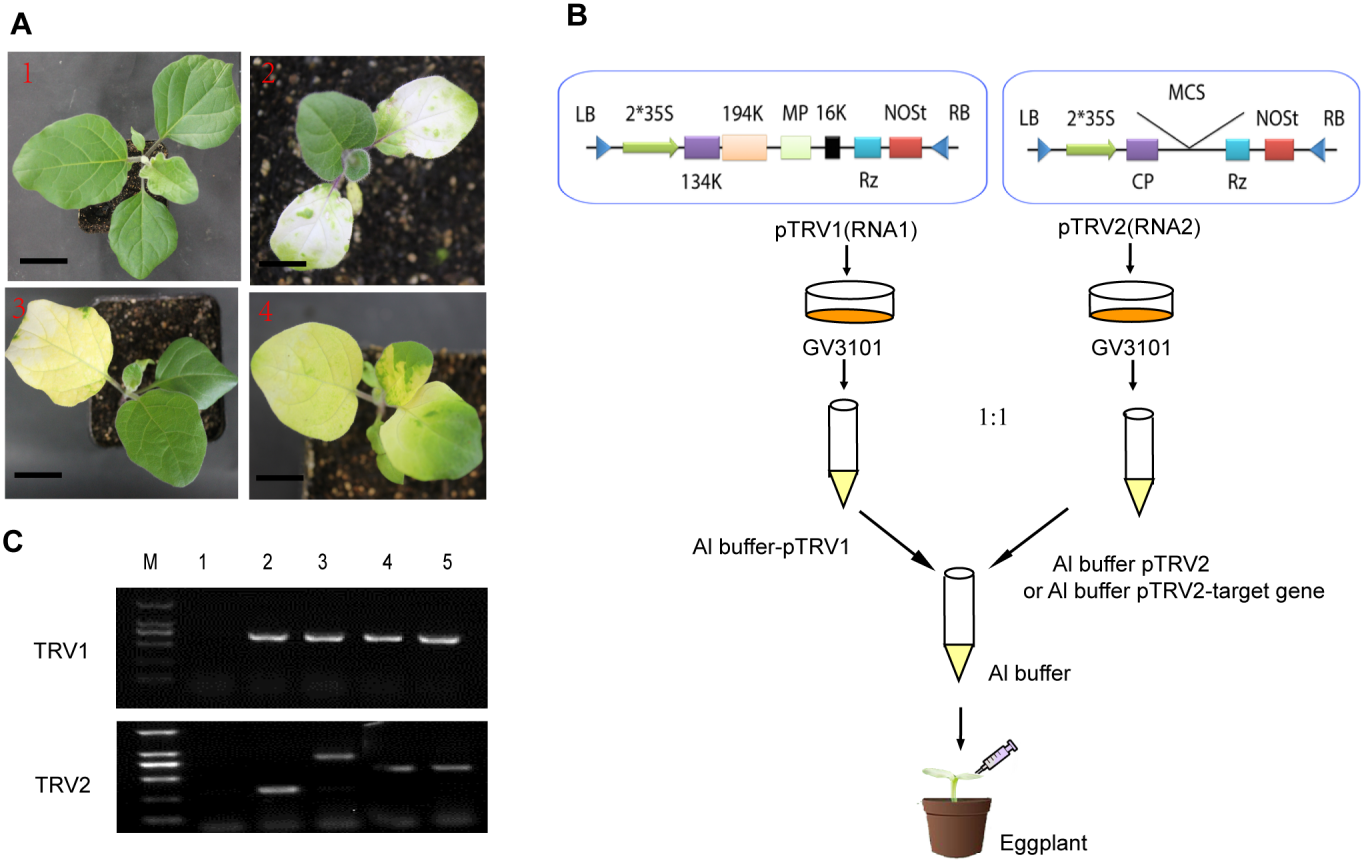
Tobacco rattle virus-mediated silencing of *PDS*, *ChlI* and *ChlH* in eggplant leaves. (A) Phenotypes of eggplant leaves infiltrated with different VIGS constructs: 1: Empty vector; 2: *TRV-PDS*, 3: *TRV-ChlI*, 4: *TRV-ChlH*. Scale bar equals 1cm. (B) Schematic depiction of virus-induced gene silencing (VIGS) treatment in eggplant (C) Confirmation of TRV infection in leaves by PCR. M, 2000 Marker; 1: Mock-infected; 2: Empty vector; 3: *TRV-PDS*, *4*: *TRV-ChlI*, *5*: *TRV-ChlH*. PDS, phytoene desaturase; CHLI, CHLH: Magnesium chelatase subunits. Oligonucleotides used are shown in Supplementary S1 Table.

VIGS was performed using Tobacco rattle virus (TRV) according to a previous study [10, 11]. *Agrobacterium* strain GV3101 containing the pTRV1, pTRV2, pTRV2-*SlPDS*, pTRV2-*SlChlI* or pTRV2-*SlChlH* vector was grown at 28°C in LB medium (pH 5.6) containing 10 mM MES and 20 μM acetosyringone and the antibiotics kanamycin, gentamycin and rifampicin. After shaking for 12 h, the cultures were harvested and resuspended in infiltration buffer (10 mM MgCl_2_, 200 μM acetosyringone, 5% sucrose) to a final OD_600_ of 2.0. Resuspensions of pTRV1 were combined with pTRV2, pTRV2-*SlPDS*, pTRV2-*SlCHLI* or pTRV2-*SlCHLH* at a 1:1 ratio and incubated at room temperature for 3 h. *Agrobacterium* was infiltrated into eggplant seedlings with a 1 ml needleless syringe (Fig 1A); seedlings infiltrated with pTRV1 and pTRV2 were used as controls. Each inoculation was carried out three times, and 20 different plants were infiltrated each time. When the VIGS phenotype was visible, Non-green leaves were collected from the plants and stored at -80°C.

### Sequence analysis of glycoalkaloid biosynthesis in eggplant

Homology searches were performed using the basic local alignment search tool (BLAST) from Sol Genomics Network (https://solgenomics.net) (Supplementary S2 Table). The conserved domain was predicted with InterPro (http://www.ebi.ac.uk/interpro/scan.html). Multiple alignments of amino acid sequences from eggplant and tomato were performed using DNAman 6.0 (Lynnon Biosoft, Canada).

### RNA extraction, reverse transcription and qRT-PCR

Total RNA was isolated from leaf samples using an RNA Prep Pure Plant kit (Tiangen, Beijing, China) containing DNase. RNA integrity was checked by agarose gel electrophoresis. RNA concentration and purity were evaluated using a NAS-99 spectrophotometer (ATCGene, NJ, USA). A 2 μg aliquot of total RNA was used for cDNA synthesis using a TransScript One-Step gDNA Removal and cDNA Synthesis SuperMix kit (Trans, Beijing, China) with random primers. Quantitative reverse-transcription PCR (qRT-PCR) was performed using SYBR Green PCR Master Mix with a CFX96 Real-time PCR System (Bio-Rad, CA, USA). The qRT-PCR conditions were as follows: 95°C for 10 min, followed by 39 cycles of 95°C for 15 s and 60°C for 30 s. Changes in the fluorescence of SYBR Green were monitored automatically in each cycle, and the threshold cycle (Ct) over the background was calculated for each reaction. Data were normalized against the endogenous *Actin* gene, and relative expression levels were measured using the 2^−ΔΔCt^ analysis method [12].

Analyses were carried out on three biological replicates, and Student’s *t*-test was used to determine the statistical significance of differences between two samples (*P<0.05; **P<0.01). Duncan’s multiple range test was used to compare three samples (**P<0.01). The oligonucleotide primers used are listed in Supplementary S1 Table.

### Quantification of carotenoid and chlorophyll levels by LC/PDA/APCI/MS analysis

Carotenoids and chlorophylls were extracted from lyophilized, homogeneously ground leaf tissue and analyzed as previously described [13, 14] with the following modifications: the elution gradient conditions were 0 to 1.2 min 95% A, 5% B; 3.5 min 80% A, 5% B; 12 min 30% A, 5% B, 65% C; 18 min 95%, 5% B. Chromatographic flux after equilibration was 0.8 mL/min and total run time was 18 minutes. Compounds were identified based on their absorbance spectra (VIS), exact mass (m/z) and specific retention time (RT), as well as co-migration with authentic standards, following a published method [13]. For quantification, all areas were normalized to the internal standard (DL-α-tocopherol acetate, Sigma-Aldrich) and to their individual molar extinction coefficients [15]. A second normalization to a set of external standards was performed to calculate errors during injection in the LC system. Carotenoid and chlorophyll levels were expressed as μg/gr of dry weight (DW).

### Semipolar metabolite profiling by LC/HESI/MS

Three milligrams of lyophilized, homogeneously ground tissue was extracted with 1 mL of 75% methanol/0.1% v/v formic acid spiked with 0.5 μg mL^-1^ formononetin (Sigma-Aldrich) as an internal standard. After vortexing for 30 s and centrifugation, 0.650 mL of epiphase was removed and transferred into filter (PTFE) vials for LC/MS analysis (Waters). The filtered extract (5 μl) was injected into the Liquid chromatography-Heated Electrospray Ionization-Mass Spectrometer (LC-HESI-MS). LC analysis was performed using a 150 x 2.0 mm C18 Luna column (Phenomenex, Macclesfield, UK) with a particle size of 2.5 μm. The total run time was 32 minutes, which was performed using an elution system running at 0.250 mL/min and consisting of A, water (0.1% formic acid, 10 pg/mL caffeine) and B, Acetonitrile: H_2_O 90:10 (0.1% formic acid, 10 pg/mL caffeine). The gradient was 0 to 0.5 min 95% A/5% B; 24 min 25% A/75% B; 26 min 95% A/5%. The MS analysis was performed using a Q Exactive Hybrid Quadrupole-Orbitrap^™^ Mass Spectrometer (ThermoFisher Scientific), with the HESI (Heated Electrospray Ionization) source operating in positive and negative ion modes. The mass spectrometer parameters were as follows: capillary temperature 250°C, sheath and auxiliary gas set at 40 and 10 units, respectively, spray voltage 3.5 kV, probe heater temperature 330°C and S-lens RF level 50 V. Metabolites were identified based on m/z accurate masses and retention times from in-house and public (e.g., Metlin, KEGG) databases. A subset of samples was authenticated using authentic standards. All chemicals and solvents used during the entire procedure were LC/MS grade (Chromasolv).

Heatmap analysis and Hierarchical Clustering Analysis (HCA) were performed using Genesis Software 1.7.7. HCA was applied to both metabolites and samples, using average linkage as the agglomeration rule.

## Results

### Virus-induced silencing of genes related to carotenoid and chlorophyll biosynthesis in eggplant leaves

**Fig 2:**
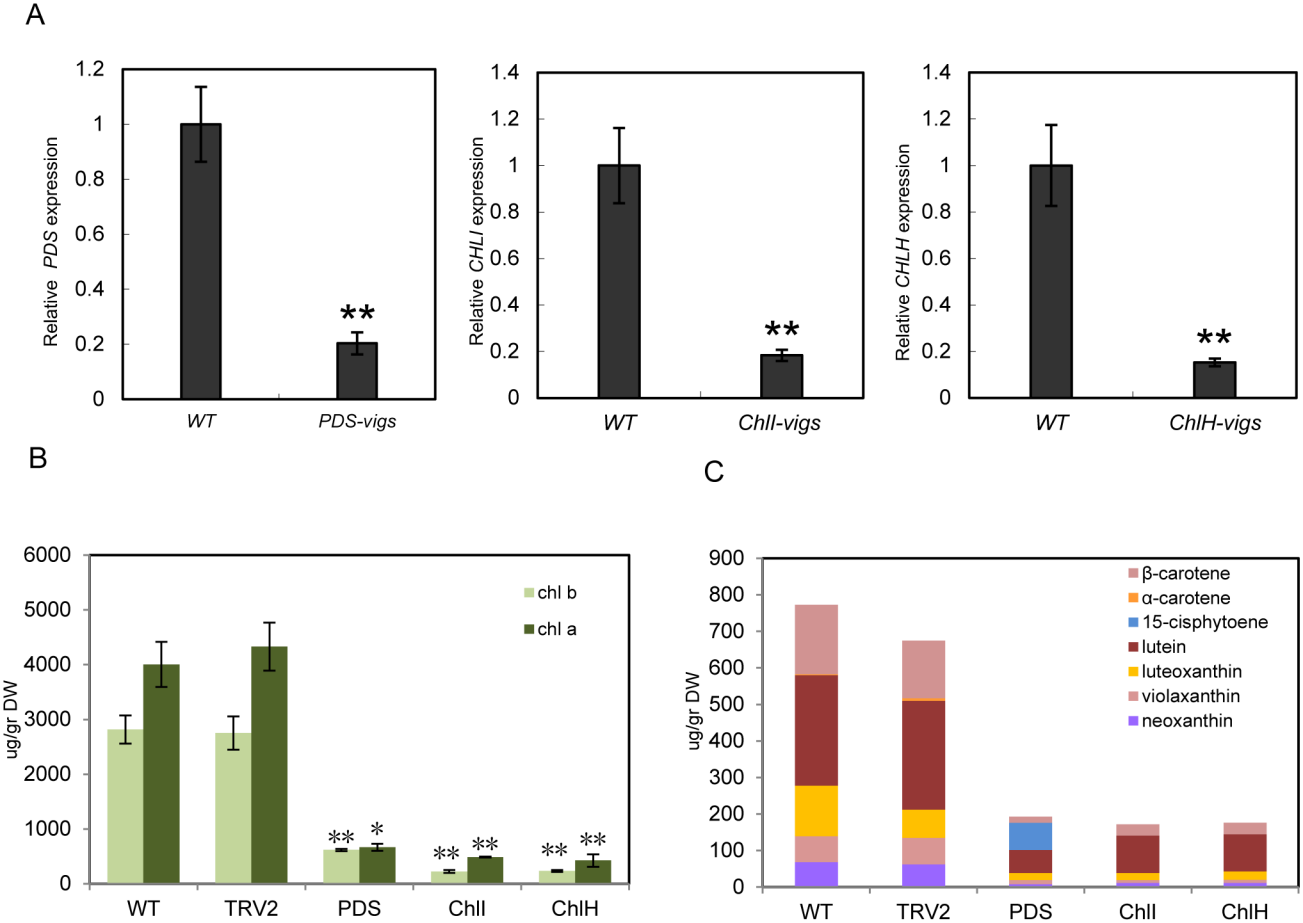
Chlorophyll and carotenoid levels are reduced in eggplant leaves with silenced *PDS*, *ChlI* and *ChlH*. (A) Quantitative RT-PCR measurement of *PDS*, *ChlI* and *ChlH* mRNA. Data are the avg. ± *SD* of three biological replicates. (B) Quantitation of chlorophyll levels in WT, *TRV*, *TRV-PDS*, *TRV-ChlI* and *TRV-ChlH* leaves. (C) Quantitation of carotenoids levels in WT, *TRV*, *TRV-PDS*, *TRV-ChlI* and *TRV-ChlH* leaves. Student’s *t*-test was used to determine the statistical significance of differences between two samples (*P<0.05; **P<0.01). Duncan’s multiple range test was used to compare three samples (**P<0.01).

To investigate the effects of the chlorophyll and carotenoid biosynthesis pathways on glycoalkaloid production in eggplant leaves, we selected three key candidate genes, *PDS*, *ChlI* and *ChlH*, for TRV-mediated VIGS to block carotenoid biosynthesis (*PDS*) or chlorophyll biosynthesis (*ChlI* and *ChlH*). We infiltrated the leaves of 2-week-old eggplant seedlings with Agrobacterium GV3101 cultures containing pTRV1- and pTRV2-candidate constructs at a 1:1 ratio using a needleless syringe (Fig 1A). Seedlings infiltrated with Agrobacterium cultures harboring pTRV1 and pTRV2 were used as a negative control. The inoculated seedlings were planted in soil and observed every 5 days under normal growth conditions. Approximately 2 weeks after inoculation, typical features of targeted gene silencing were observed, such as photobleaching due to *PDS* silencing and yellowing due to *ChlH* and *ChlI* silencing, whereas the control plants remained green (Fig 1B). Moreover, as the cultivation time increased, the silenced leaf phenotype was observed throughout the leaves of the silenced plants, indicating that VIGS can be used effectively to silence target genes in eggplant. To investigate the effects of TRV infection and silencing of target genes at the molecular level, we used RNA from leaves collected from silenced and control plants to investigate viral and target gene expression levels via quantitative RT-PCR. We used primers that anneal to the outside region of the *PDS*, *ChlI and ChlH* genes targeted for silencing, and two strands of the TRV virus were detected. All silenced and TRV-infected leaves showed the presence of both the TRV1 and TRV2 genomes (Fig 1C). The reduction in *PDS*, *ChlI* and *ChlH* mRNA levels ranged from 80 to 85% (Fig 2A).

To further investigate the effects of silencing the three target genes on chlorophyll and carotenoid biosynthesis at the metabolic level, we measured the contents of carotenoids and chlorophylls in the silenced and control plants by LC/PDA/APCI/MS analysis. Chlorophyll levels were reduced by 84% in *TRV-PDS*-infected leaves and by 89–90% in *TRV*-*ChlI* and *TRV*-*ChlH*-silenced leaves (Fig 2B). Carotenoid levels were respectively reduced by approximately 72–75% in the TRV-*PDS/ChlI/ChlH*-silenced samples compared to the mock-infected control. With the exception of 15-*cis*-phytoene, which was the most abundant compound in *TRV-PDS*-silenced leaves (comprising 38% of total carotenoid contents), the distribution pattern of carotenoids was similar in mock-infected versus silenced leaves, including the relative levels of lutein, luteoxanthin, *β*-carotene and *β*-xanthophylls (Fig 2C). These results indicate that the silencing efficiency of these genes is high and silencing of *PDS*, *ChlI* and *ChlH* successfully inhibited carotenoid and chlorophyll biosynthesis in eggplant leaves. The silenced lines were effective research materials to study the relationship between chlorophyll, carotenoid and glycoalkaloid biosynthesis.

### Blocking phytochromes biosynthesis reduces SGA accumulation in eggplant leaves

**Fig 3:**
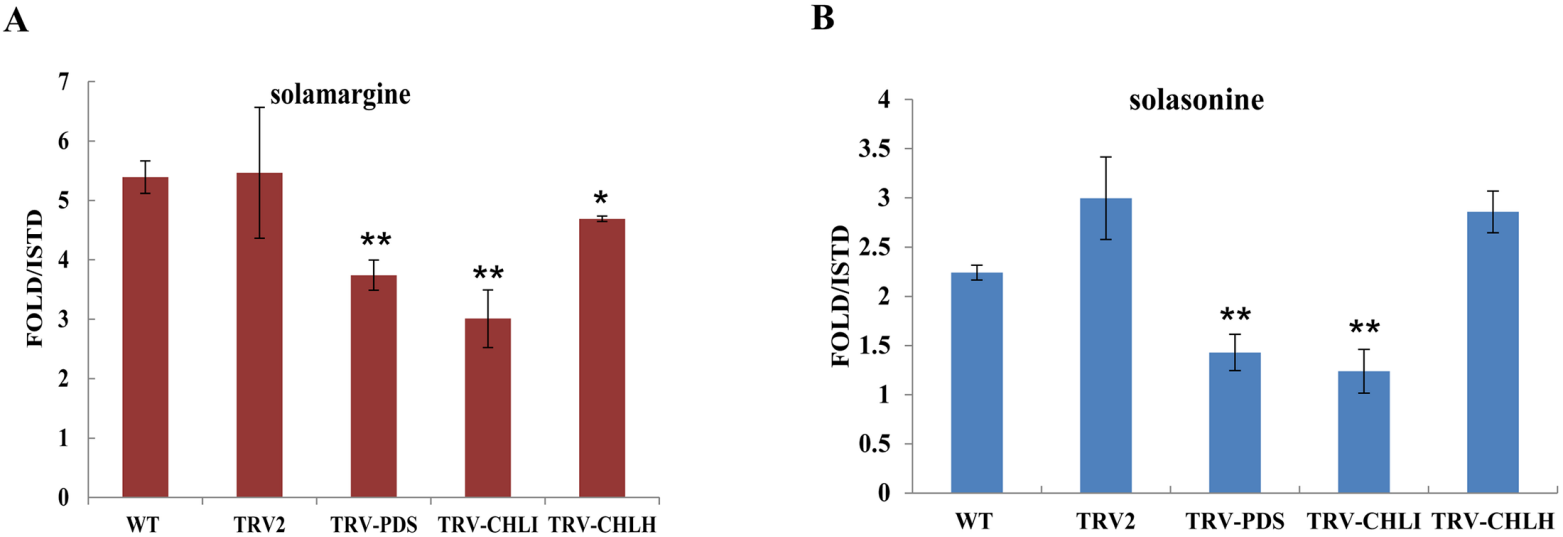
Suppression of carotenoid and chlorophyll levels decrease glycoalkaloid biosynthesis in eggplant leaves. Relative content of solamargine (A) and solasonine (B) in eggplant leaves mock-infected and infected with *TRV*, *TRV-PDS*, *TRV-ChlI* and *TRV-ChlH*. ISTD means Internal Standard, because metabolites have been relatively quantified using Formononetin as internal standard. Data are the avg. ± *SD* of three biological replicates, and Student’s *t*-test was used to determine the statistical significance of differences between two samples (*P<0.05; **P<0.01). Duncan’s multiple range test was used to compare three samples (**P<0.01).

**Fig 4:**
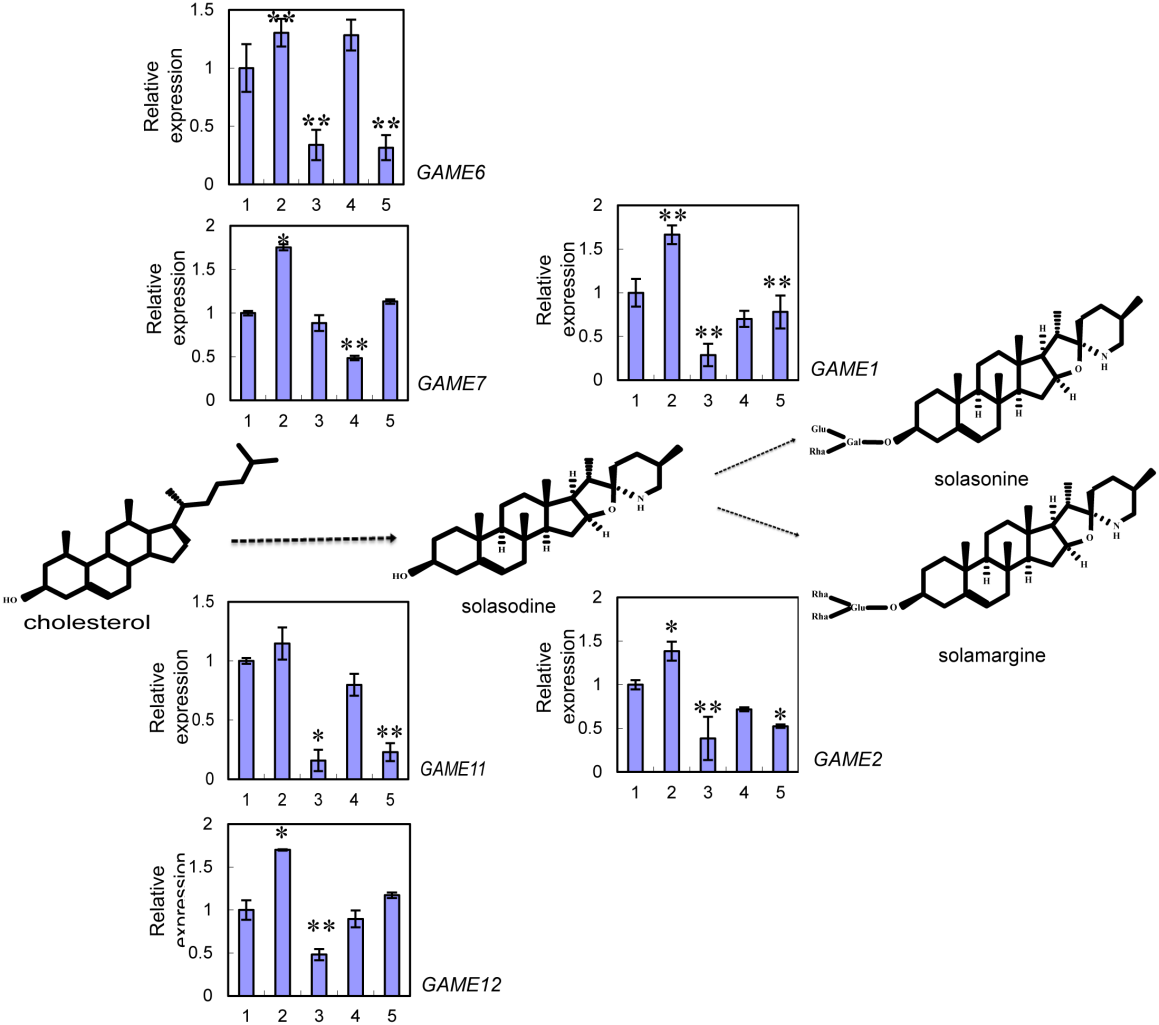
Relative expression levels of glycoalkaloid pathway genes in leaves infected with different constructs. A schematic view of the SGA biosynthetic pathways. Dashed arrows represent multiple biosynthetic reactions. Graphs next to each gene name show expression levels determined by qRT-PCR. Expression levels were normalized to the *Actin* housekeeping gene and to the mock-infected control. The X-axis number can be expressed as follows, 1: Mock-infected; 2: Empty vector; 3: *PDS*-silenced, *4*: *ChlI*-silenced, *5*: *ChlH*-silenced. Data are the avg. ± *SD* of three biological replicates. Asterisks indicate a significant difference from the control in a Student’s *t*-test (**P < *0*.*01* and *P < *0*.*05*).

Since the production of chlorophylls and carotenoids in eggplant leaves was successfully reduced by silencing *PDS*, *ChlI* and *ChlH*, we measured the glycoalkaloid contents in the silenced plants versus the control samples via LC/HESI/MS. Compared to the control, the levels of both solamargine and solasonine were strongly decreased in leaves infected with *TRV-PDS* (solasonine reduced by 53%; solamargine reduced by 33%) and *TRV-ChlI* (solasonine reduced by 59%; solamargine reduced by 46%). However, the levels of these compounds in leaves infected with *TRV*-*ChlH* were decreased only slightly (5–15%) compared to leaves infected with TRV alone (Fig 3, S3 Table). To further explore the decrease in SGA levels in the silenced eggplant leaves at the molecular level, we measured the transcript levels of genes involved in biosynthesis process from cholesterol to sterol glycoalkaloids, including *GAME1*, *2*, *6*, *7*, *11* and *12*. The *GLYCOALKALOID METABOLISM* (*GAME*) genes, located close to each other on the genome and organized in a metabolic gene cluster, take part in the primary pathway synthesizing SGAs. Genes encoding cytochrome P450s (*GAME7* and *GAME6* located on chromosome 7 in a cluster), and a dioxygenase (*GAME11* on chromosome 7) are involved in the hydroxylation and oxidation of the cholesterol skeleton, while a transaminase protein (GAME12 on chromosome 12) incorporates the nitrogen atom into the SA aglycone. Finally, the glycosyltransferases (*GAME1*, *GAME2* on chromosome 7) add the sugar moieties to aglycone to form glycoalkaloid [5]. The mRNA levels of these *GAME* genes were reduced by approximately 50–86% in *PDS*-silenced plants, approximately 32–81% in *ChlH*-silenced plants and approximately 47–77% in *ChlI*-silenced plants compared to the TRV-infected control (Fig 4). These results indicate that chlorophyll and carotene biosynthesis affects the accumulation of glycoalkaloids in eggplant leaves.

### Semipolar metabolite profiling of *PDS*-, *ChlI*- and *ChlH*-silenced plants by LC-HESI-HRMS

**Fig 5:**
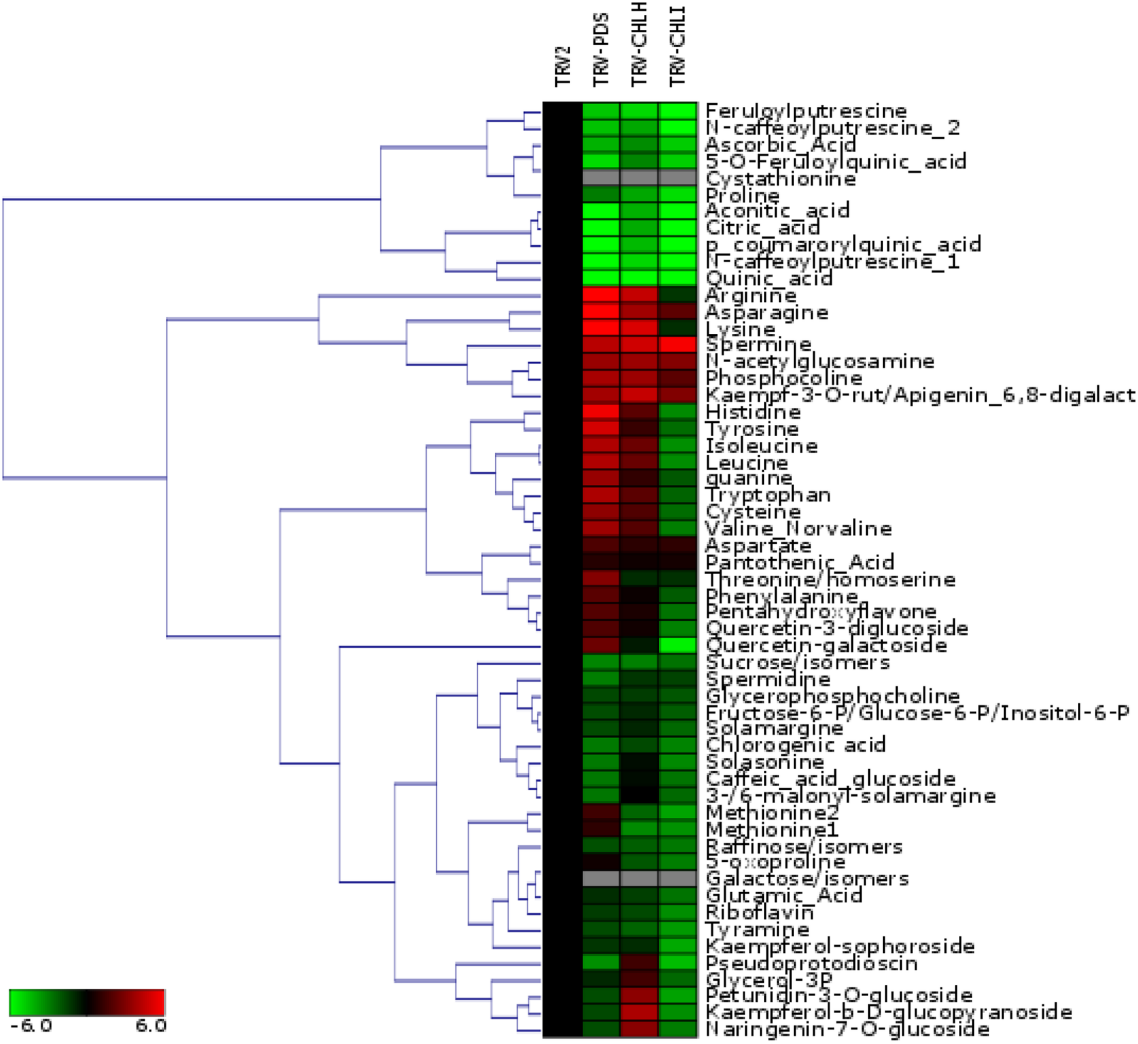
Metabolic analysis by LC-HESI-MS. Heatmap of semi-polar metabolites (including glycoalkaloids) profiled in eggplant leaves. Colors represent fold-change values between each line and the control (TRV) sample. Red and green indicate up- and down-regulated metabolites, respectively. Fold-change values were log2-transformed. Both columns (samples) and rows (metabolites) were subjected to hierarchical clustering analysis (HCA).

To assess the metabolic consequences of silencing *PDS*, *ChlI* and *ChlH* in eggplant leaves, we measured the levels of 58 semipolar metabolites, including glycoalkaloids, amino acids, phenylpropanoids, carboxylic acids, sugars and vitamins, via liquid chromatography-heated electrospray ionization-high resolution mass spectrometry (LC-HESI-HRMS). To evaluate the differences in the silenced leaves compared to the mock-infected control, we expressed the relative intensities of semi-polar metabolites in terms of the fold-change in the level of each compound between each silenced line and the TRV control (Fig 5). We subjected both the metabolites and samples to Hierarchical Clustering Analysis (HCA). The silenced plants were separated into two different clusters: *TRV*-*ChlH*, *TRV*-*PDS* and TRV-*ChlI* versus *TRV*. Some of the most dramatic changes in terms of metabolic distribution pertained to glycoalkaloids; the levels of solamargine, 3-/6-malonylsolamargine and solasonine were lower in *TRV*-*PDS*, *TRV*-*ChlH* and *TRV*-*ChlI* silenced leaves compared to the control. The levels of sugars such as raffinose and sucrose (or isomers maltose / isomaltose / trehalose), as well as their phosphorylated derivatives (fructose-6P/glucose-6P /inositol-6P), were lower in the silenced samples than in the control, while fructose (or isomers galactose/glucose/inosositol) was not detectable in silenced leaves. The levels of aconitate and citrate, which are both involved in the tricarboxylic acid cycle (TCA) and were grouped by the HCA, were lower in silenced leaves than in the control. Finally, the levels of polyphenols such as chlorogenic acid, quinic acid and their derivatives (feruloyl-/coumaroyl-quinic acid), as well as several polyamine conjugates (caffeoylputrescine and feruloylputrescine), were lower in silenced leaves than in the control. Several differences in the distribution of these metabolites differentiated *TRV*-*ChlI* plants from the other silenced plants in the HCA. Phenylalanine, tyrosine and tryptophan, as well as several other amino acids (cysteine, leucine, isoleucine and histidine), which were grouped by HCA, were present at higher levels in *TRV*-*ChlH* and *TRV*-*PDS* plants but at much lower levels in *TRV*-*ChlI* plants compared to the control. Conversely, methionine (isomer 1 and 2) levels were lower in both *TRV*-*ChlH* and *TRV*-*ChlI* plants but higher in *TRV*-*PDS* plants compared to the control. Surprisingly, cystathionine, the upstream precursor of methionine, accumulated in control leaves but was not detectable in any of the silenced samples. Cystathionine is involved in ethylene and glucosinolate biosynthesis [16]. The metabolomics data suggests that there is a difference in the specific mechanism of affecting glycoalkaloids biosynthesis between silencing magnesium chelatase and silencing phytoene desaturase.

## Discussion

Steroidal glycoalkaloids (SGAs) are nitrogen-containing compounds produced primarily by liliaceous and solanaceous species [17]. SGAs are stored in all plant tissues, including roots, flowers, leaves, fruits [1, 6, 18, 19]. SGAs act as phytoanticipins, providing the plant with a pre-existing defence against a broad range of pathogens, when agro-infiltration, SGA as a protector against *Agrobacterium* invasion, so the SGA biosynthesis-related gene expression and SGA content increased (Figs 3 and 4). Considering a large number of regulated steps and the differential regulation observed as mentioned, I cannot exclude the possibilities of the involvement of additional mechanisms, such as indirect regulation through other transcription factors or metabolite-mediated feedback regulation, which would explain why *ChlI* and *ChlH*-silencing activated the expression of some *GAME* genes, such as *GAME6*,*GAME12* (Fig 4).

To date, most research on SGAs has focused on elucidating their structure, characterizing their composition in different species and unraveling their biosynthetic pathway [4, 5, 18, 20–26]. Many studies have shown that light influences the production of chlorophyll and alkaloids. The effects of different post-harvest light regimens on the contents of the two most important glycoalkaloids (*α*-chaconine and *α*-solanine) in tubers were found to differ among potato varieties [27]. In addition, light has been shown to alter the accumulation of both chlorophyll and SGAs in potato, with their levels responding similarly to certain light sources [28]. In a previously reported *pds3* mutant (defective in chlorophyll biosynthesis) reduced expression of *IPI* and *GGPS* impairs the MVA metabolic pathway, thereby decreasing the production of cholesterol, which is the precursor of SGAs [29]. Although some studies have indicated that chlorophyll contents are not related to glycoalkaloid levels in plants [7, 30–32], others have shown that chlorophyll biosynthesis is involved in the biosynthesis of glycoalkaloids, and that formate, glycine and pyruvate are intermediates in the biosynthesis of mevalonate and (subsequently) solanidine, the immediate precursor of the SGAs *α*-chaconine and *α*-solanine [31, 33–35]. These apparently contradicting results prompted us to explore the relationship between alkaloid biosynthesis and chlorophyll accumulation.

In this work, we provide evidence that steroidal glycoalkaloid biosynthesis in eggplant leaves depends on photosynthetic pigment accumulation. Upon the reduction of chlorophyll and carotenoid biosynthesis in eggplant via VIGS targeting biosynthetic genes (*PDS*, *ChlI* and *ChlH*), we detected a decrease in glycoalkaloid contents along with decreases in both chlorophylls and carotenoids (Figs 2 and 3). This discovery not only provides important insight into the SGA biosynthetic pathway in eggplant but also lays the foundation for reducing the levels of endogenous antinutritional compounds in crops.

The LC-HESI-HRMS method was successfully applied to the metabolite analysis in eggplant. We found that cystathionine accumulated in control leaves but was not detectable in the silenced plants. Notably, cystathionine is the precursor of methionine. Indeed, methionine 1 and methionine 2 levels were significantly reduced in *ChlI*-VIGS and *ChlH*-VIGS eggplant leaves compared to the control. This reduction is particularly intriguing because methoinine is involved in ethylene biosynthesis and ethylene has been shown to induce glycoalkaloid biosynthesis. In tomato fruit, *GLYCOALKALOID METABOLISM1 (GAME1)* expression is negatively regulated by the ethylene-signaling cascade [36, 37]. In addition, knockdown and overexpression of the APETALA2/Ethylene response factor gene, *GAME9*, in tomato and potato altered the expression of SGA-related genes and other genes in the upstream mevalonate pathway [21].Maybe ethylene is the potential link between SGAs biosynthesis and photosynthetic pigment accumulation.

In conclusion, SGAs, a class of antinutritional substances, are present in widely used food crops such as potato, tomato and eggplant [1], but cause gastrointestinal and neurological disorders and can be lethal to humans at high concentrations [38]. Therefore, decreasing the content of these unsafe compounds is important for human health. Our results indicating a link between production of photosynthetic pigments and that of SGAs provide a basis for reducing the content of endogenous anti-nutritional compounds in crops.

## Supporting information

S1 Fig Structures of potato, tomato and eggplant glycoalkaloids. (TIF)

S2 Fig Simplified view of cholesterol, carotenoid and chlorophyll biosynthesis in Solanaceae plants. Dashed arrows denote several steps and the double arrow between the cytosol and plastid indicates metabolic crosstalk between the compartments.
(TIF)

S1 Table List of primers used in this study. The first six primers were used for cloning *PDS*, *ChlI* and *ChlH* from tomato (*Solanum lycopersicum*) into the pTRV2 vector, and all other primers were used for qRT-PCR.
(TIF)

S2 Table Gene IDs of the sequences used for qRT-PCR. (TIF)

S3 Table Identification of glycoalkaloids in eggplant (LC/MS analysis). (TIF)
