# Supplementary figures and images for: The role of phytochromes in regulating biosynthesis of sterol glycoalkaloid in eggplant leaves
Source: PLoS One. 2017 Dec 13;12(12):e0189481. doi: 10.1371/journal.pone.0189481 (PMC5728552; doi:10.1371/journal.pone.0189481)

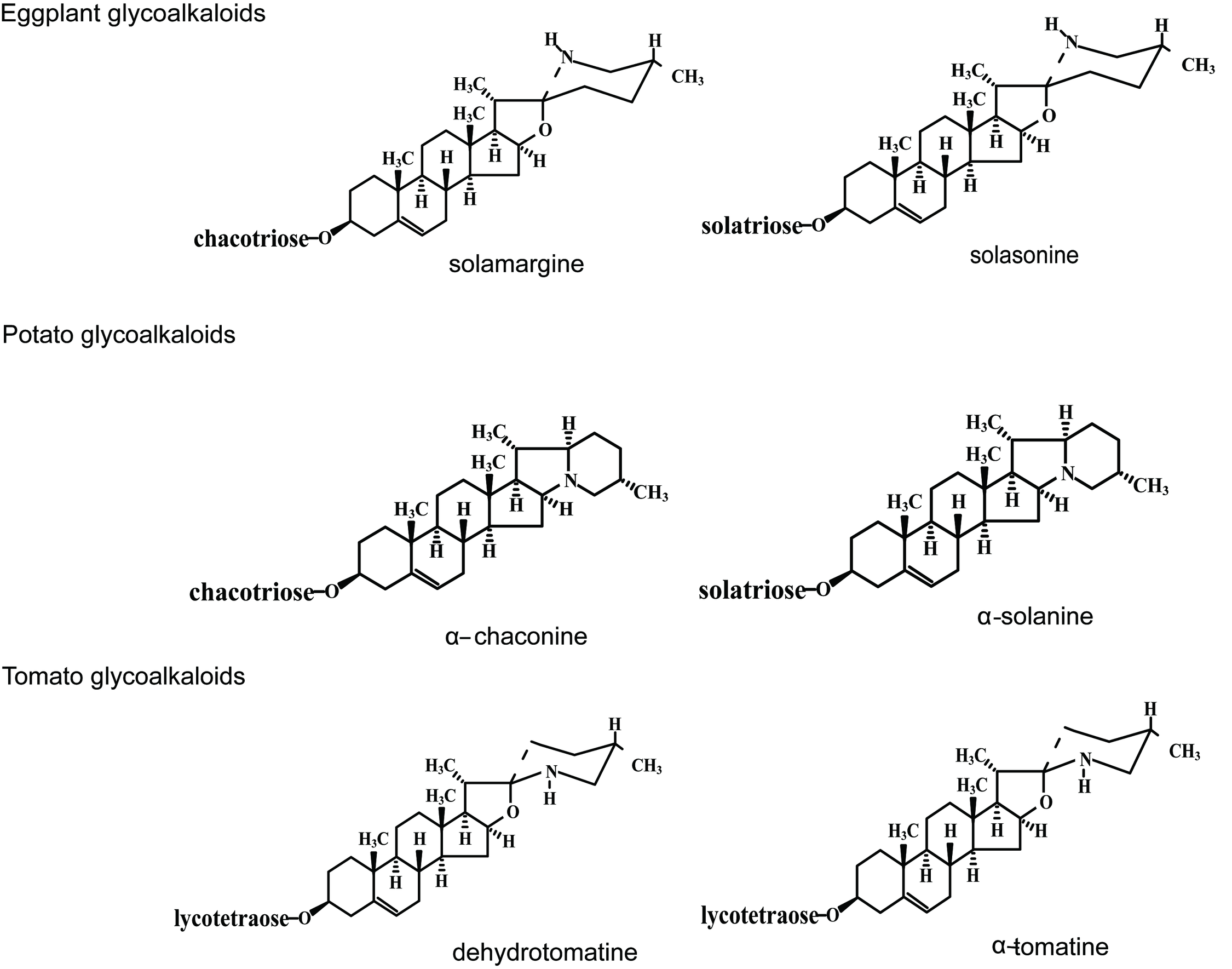

Supplement: S1 Fig — (TIF) [file pone.0189481.s001.tif]

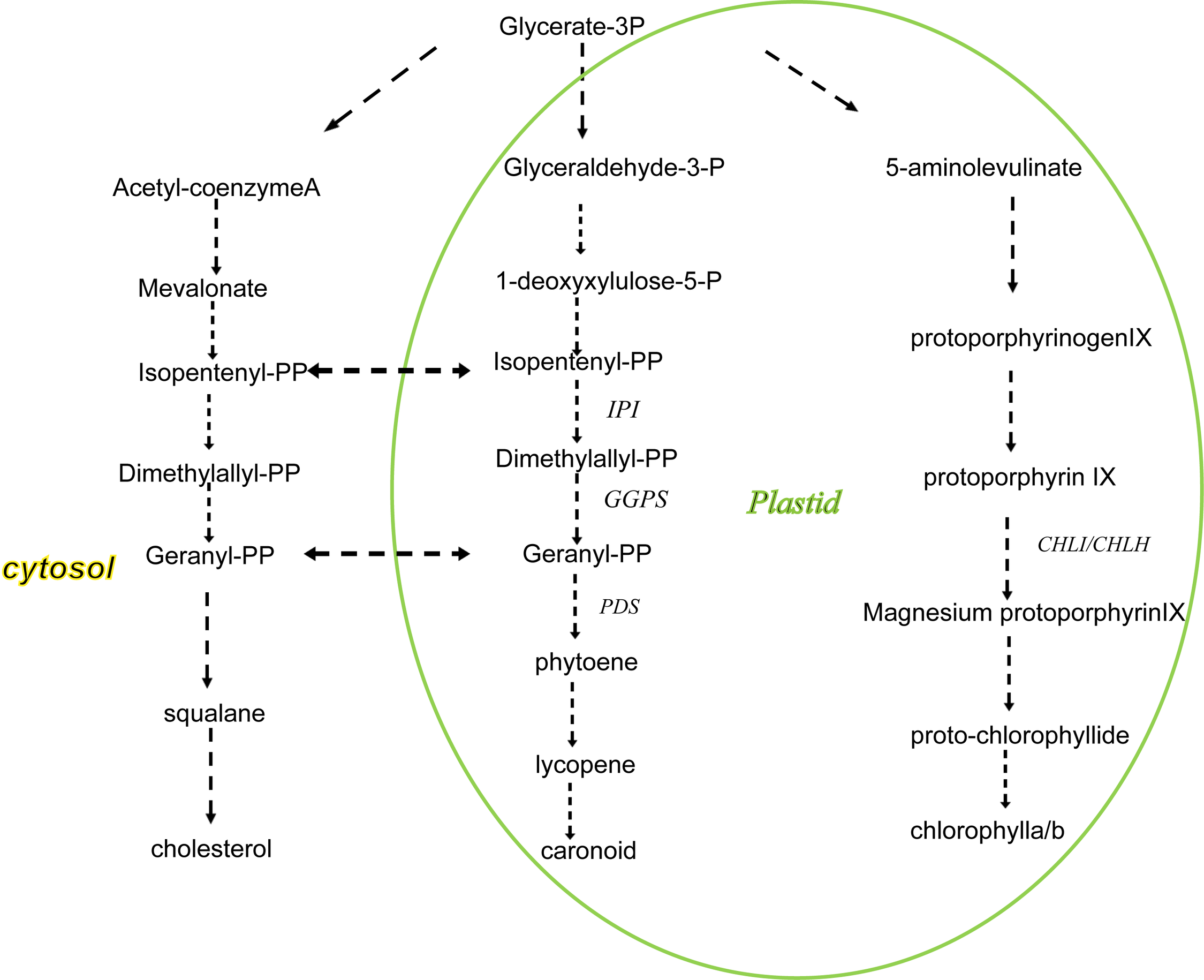

Supplement: S2 Fig — Dashed arrows denote several steps and the double arrow between the cytosol and plastid indicates metabolic crosstalk between the compartments. (TIF) [file pone.0189481.s002.tif]

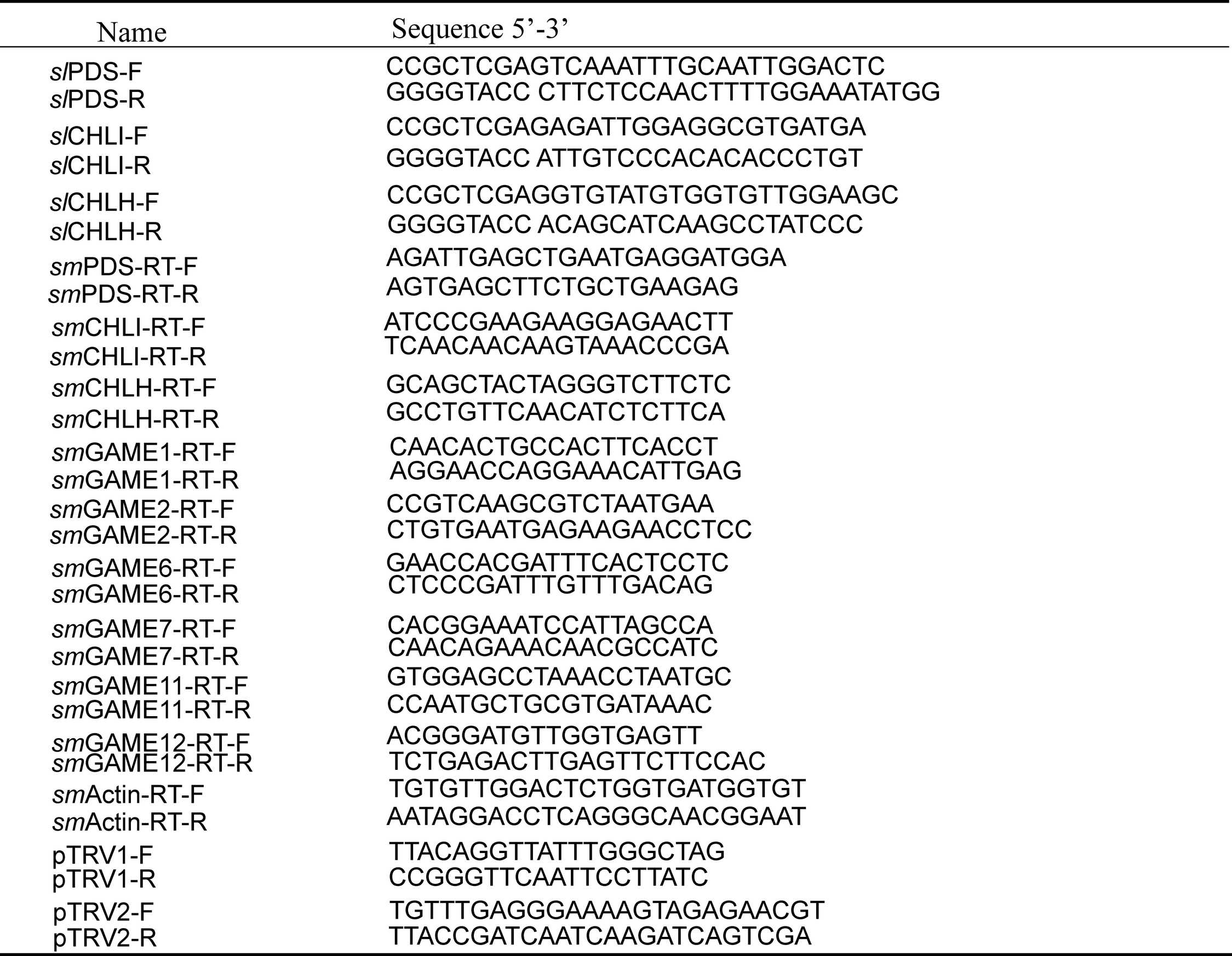

Supplement: S1 Table — The first six primers were used for cloning PDS, ChlI and ChlH from tomato (Solanum lycopersicum) into the pTRV2 vector, and all other primers were used for qRT-PCR. (TIF) [file pone.0189481.s003.tif]

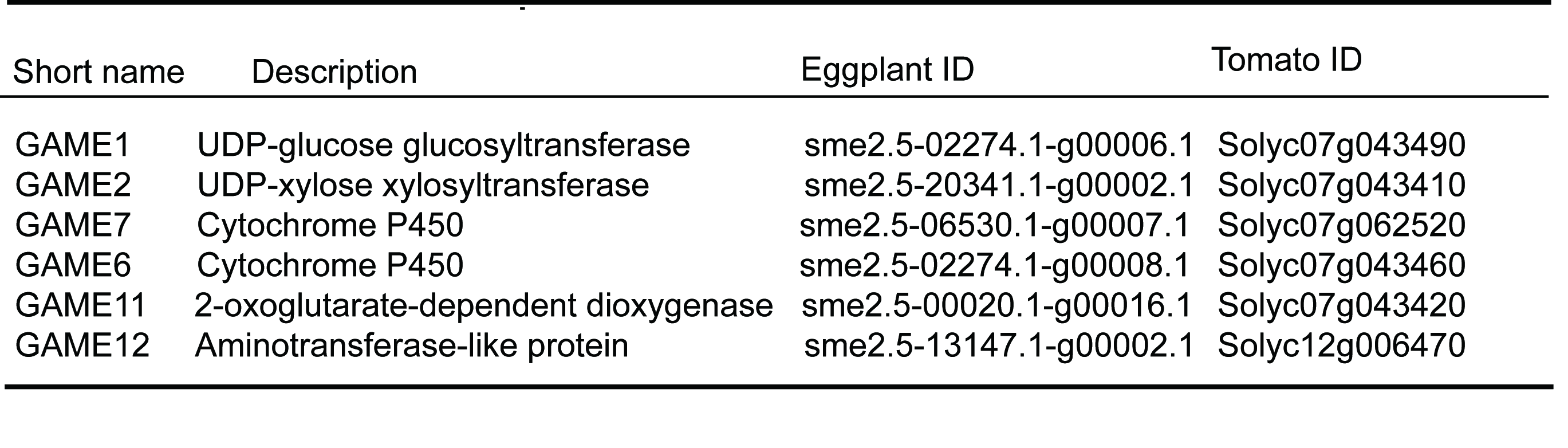

Supplement: S2 Table — (TIF) [file pone.0189481.s004.tif]

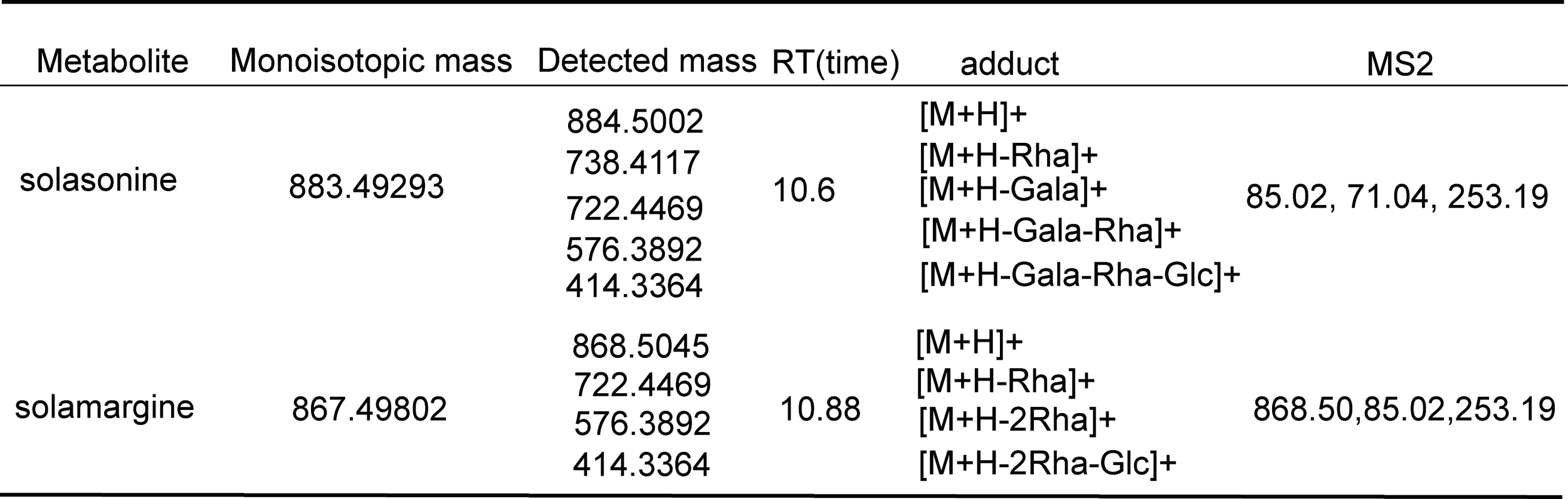

Supplement: S3 Table — (TIF) [file pone.0189481.s005.tif]
